# Relationships between root exudation and root morphological and architectural traits vary with growing season

**DOI:** 10.1093/treephys/tpad118

**Published:** 2023-09-21

**Authors:** Yuqiu Gao, Huimin Wang, Fengting Yang, Xiaoqin Dai, Shengwang Meng, Mingyuan Hu, Liang Kou, Xiaoli Fu

**Affiliations:** Qianyanzhou Ecological Research Station, Key Laboratory of Ecosystem Network Observation and Modeling, Institute of Geographic Sciences and Natural Resources Research, Chinese Academy of Sciences, 11A, Datun Road, Chaoyang District, Beijing 100101, China; School of Water Conservancy and Environment, University of Jinan, No. 336 West Nanxinzhuang Road, Shizhong District, Jinan 250022, China; Qianyanzhou Ecological Research Station, Key Laboratory of Ecosystem Network Observation and Modeling, Institute of Geographic Sciences and Natural Resources Research, Chinese Academy of Sciences, 11A, Datun Road, Chaoyang District, Beijing 100101, China; College of Resources and Environment, University of Chinese Academy of Sciences, No. 80 Zhongguancun East Road, Haidian District, Beijing 100190, China; Qianyanzhou Ecological Research Station, Key Laboratory of Ecosystem Network Observation and Modeling, Institute of Geographic Sciences and Natural Resources Research, Chinese Academy of Sciences, 11A, Datun Road, Chaoyang District, Beijing 100101, China; College of Resources and Environment, University of Chinese Academy of Sciences, No. 80 Zhongguancun East Road, Haidian District, Beijing 100190, China; Qianyanzhou Ecological Research Station, Key Laboratory of Ecosystem Network Observation and Modeling, Institute of Geographic Sciences and Natural Resources Research, Chinese Academy of Sciences, 11A, Datun Road, Chaoyang District, Beijing 100101, China; College of Resources and Environment, University of Chinese Academy of Sciences, No. 80 Zhongguancun East Road, Haidian District, Beijing 100190, China; Qianyanzhou Ecological Research Station, Key Laboratory of Ecosystem Network Observation and Modeling, Institute of Geographic Sciences and Natural Resources Research, Chinese Academy of Sciences, 11A, Datun Road, Chaoyang District, Beijing 100101, China; College of Resources and Environment, University of Chinese Academy of Sciences, No. 80 Zhongguancun East Road, Haidian District, Beijing 100190, China; Qianyanzhou Ecological Research Station, Key Laboratory of Ecosystem Network Observation and Modeling, Institute of Geographic Sciences and Natural Resources Research, Chinese Academy of Sciences, 11A, Datun Road, Chaoyang District, Beijing 100101, China; College of Resources and Environment, University of Chinese Academy of Sciences, No. 80 Zhongguancun East Road, Haidian District, Beijing 100190, China; Qianyanzhou Ecological Research Station, Key Laboratory of Ecosystem Network Observation and Modeling, Institute of Geographic Sciences and Natural Resources Research, Chinese Academy of Sciences, 11A, Datun Road, Chaoyang District, Beijing 100101, China; College of Resources and Environment, University of Chinese Academy of Sciences, No. 80 Zhongguancun East Road, Haidian District, Beijing 100190, China; Qianyanzhou Ecological Research Station, Key Laboratory of Ecosystem Network Observation and Modeling, Institute of Geographic Sciences and Natural Resources Research, Chinese Academy of Sciences, 11A, Datun Road, Chaoyang District, Beijing 100101, China; College of Resources and Environment, University of Chinese Academy of Sciences, No. 80 Zhongguancun East Road, Haidian District, Beijing 100190, China

**Keywords:** absorptive fine roots, nutrient acquisition strategy, root exudates, root functional traits, root physiological trait, seasonal variation

## Abstract

Plants allocate a substantial amount of C belowground for root exudates and for the construction and adjustment of root morphological and architectural traits. What relationships exist between root exudates and other root traits and these relationships change with growing season, however, remain unclear. We quantified the root exudation rate and root morphological traits, including total root length (RL), total root surface area (RS), root diameter (RD), specific root length (SRL), specific root area (SRA) and root tissue density (RTD), and architectural traits, such as branching intensity (BI), and investigated their associations during the rapidly growing season (April and August) and the slowly growing season (December) of three common native tree species, *Liquidambar formosana*, *Michelia maudiae* and *Schima superba*, in subtropical China. We found that the linkages of RD, SRL, SRA, RTD and BI did not change with the growing season, reflecting their highly conservative relationships. The root exudation rate varied significantly with growing season (*P* < 0.05) and produced various associations with other root traits at different growing seasons. During the rapidly growing season (i.e., April), the exudation rate was the highest and was positively correlated with RL. The exudation rate was the lowest during the slowly growing season (i.e., December) and was negatively associated with RL, RS and RTD. Our findings demonstrate the seasonality of the linkages of root exudation rate with other root traits, which highlights the highly plastic and complex associations of belowground root traits. These findings help to deepen our understanding of plant nutrient acquisition strategies.

## Introduction

Absorptive roots play an important role in plants, foraging for sufficient water and mineral nutrients from soil (McCormack et al. 2015, Freschet et al. 2017). Plants generally increase resource acquisition via increasing total root length (RL) to exploit a larger volume of soil and to occupy resource patches (Lõhmus et al. 2006, Ostonen et al. 2011) or to adjust root morphological and architectural functional traits (e.g., higher specific root length, SRL) to improve the nutrient absorption efficiency (Pregitzer et al. 2002, Ostonen et al. 2011, 2017, Liu et al. 2015). However, whether root morphological and architectural traits change or not, resource availability is a prerequisite for root nutrient absorption. Root exudation, as a key root physiological trait (Bardgett et al. 2014, Laliberte 2017), is important for nutrient availability because it supplies labile carbon (C) to microbes, which can promote microbial decomposition (Phillips et al. 2008, Tückmantel et al. 2017, Vives-Peris et al. 2020). Additionally, special components (such as carboxylates or enzymes) of root exudates also can directly affect the soil nutrient availability by mobilizing sparingly soluble inorganic and organic nutrients (Wen et al. 2019) and by changing soil pH (Vives-Peris et al. 2020). Root exudation, therefore, is also an effective strategy for nutrient acquisition (Roumet et al. 2016, Laliberte 2017).

Root exudates come from the assimilated carbohydrates of plants and can consume up to one-third of the total photosynthates (Liese et al. 2018). A widely accepted mechanism for the control of root exudates is C as the ‘currency’ to trade for nutrients (Prescott et al. 2020). However, plants not only invest a large amount of C in the form of root exudates belowground for nutrient exchange (Phillips et al. 2008) but also allocate a substantial amount of C for root construction and maintenance (Eissenstat and Yanai 1997). For an individual plant, investing available carbohydrates into one trait would directly constrain the allocation of remaining resources to other traits (Eichenberg et al. 2015). Thus, it is inevitable that plants coordinate their C allocation belowground for root exudates, root morphological and architectural traits (Lambers et al. 2006, Lynch 2015, Wen et al. 2019), especially when C is limited. Despite the widespread recognition of root exudates in nutrient acquisition, little is known about the relationships of root exudation with root morphological and architectural traits (Oburger and Jones 2018). Studies on grassland species and crop species reveal that root exudation rate increases with increasing root diameter (RD) but decreases with increasing SRL (Wen et al. 2019, Williams et al. 2021). The findings for woody species show no correlation or a positive linkage between root exudation rate with SRL and show a significant positive correlation with specific root area (SRA) and a negative correlation with root tissue density (RTD) (Meier et al. 2020, Sun et al. 2021). The little and inconsistent evidence reflects the poor understanding of the relationships of root exudation with root morphological and architectural traits. What exactly the relationships are among root exudates with other root traits remain unclear.

Previous studies indicate that RD, SRL, RTD and root branching intensity (BI) vary greatly among different seasons (Liu et al. 2020). Root exudation also varies among seasons (Liese et al. 2018, Jakoby et al. 2020, He et al. 2021), and the highest exudation rate is expected at the onset of the growth stage (Phillips et al. 2008). Plants modify many root traits to adapt to limited and various resources (Wang et al. 2016, Valverde-Barrantes et al. 2017, Ma et al. 2018). Additionally, plant nutrient requirements also vary among seasons (Liu et al. 2020). Specifically, plants may preferentially allocate more C for root proliferation and construction to occupy resource, rapidly at the early growing season (Broeckx et al. 2013, Cao et al. 2020), and the highest exudation rate is expected to meet the plant's higher nutrient requirements at the onset of the growth stage (Phillips et al. 2008). Thus, the physiological and other traits may be coordinated to enhance nutrient acquisition. During the slowly growing season, plant would invest lower C for root construction but would retain root maintenance because of the environmental conditions unsuitable for plant growth, such as low temperature and drought (Eissenstat and Yanai 1997, Alvarez-Uria and Körner 2007, Sebastian et al. 2015). Nevertheless, most studies have focused almost entirely on independent changes of root traits and have paid less attention to how associations among root traits vary with growing season. How the relationships between root exudation, root morphological and architectural traits change with plant growing season is still unclear.

*Cunninghamia lanceolata*, *Pinus massoniana* and *Pinus elliottii* pure coniferous plantations are extensively distributed in subtropical China. However, the pure plantations have created a series of ecological problems, such as low diversity and ecosystem stability (He et al. 2013, Wang et al. 2013). Mixing broad-leaved trees in the conifer plantations is the most popular method to resolve these ecological problems of monospecific plantations (Huang et al. 2018). As dominant species in the broad-leaved forest in subtropical China, *Liquidambar formosana*, *Michelia maudiae* and *Schima superba* are generally selected to be planted in the pure coniferous forest according to practical experiences. Identifying the variations in root traits and their linkages would help us understand the resource acquisition strategies of species mixed in plantations and thus manage them effectively. We designed an experiment to quantify root exudation and root morphological and architectural traits during different growing seasons for the three typical broad-leaf trees. The similar condition (age, soil condition and mycorrhizal type) of these three target tree species can ensure to better reflect the effect of growing season on root traits and their linkages. Our objectives are to (i) investigate the variation in root exudation rate in different growing seasons; (ii) identify the relationships among root exudation with root morphological and architectural traits; and (iii) clarify the response of the relationships to plant growing season. We hypothesized that plants would invest more C in root growth by root construction, adjustment and exudates to meet nutrient demands during the rapidly growing season, whereas they would reduce the investment in exudates and would preferentially maintain root systems during the slowly growing season; therefore, the relationships of root morphological and architectural traits with root exudates would be coordinated during the rapidly growing season when photosynthate is sufficient, but there would be a trade-off during the slowly growing season because C is limited.

## Materials and methods

### Study site and experimental design

The study was set up at the Qianyanzhou Ecological Research Station (26°44′48″N, 115°04′13″E) in Taihe County, Jiangxi Province, China. The site experiences a subtropical monsoon climate with a mean annual temperature of 17.9 °C and precipitation of 1489 mm. In April, August and December 2019, the average temperature and precipitation were 20.2, 29.2 and 9.1 °C, and 207.2, 45.4 and 49.6 mm, respectively, which causes plants to grow all year round (Table S1 available as Supplementary data at *Tree Physiology* Online). The soils are weathered from red sandstone and mudstone and are classified as a Typic Dystrudept in the USDA classification.

Pure coniferous plantations were extensively established by planting *P. massoniana*, *P. elliottii* and *C. lanceolata* between 1984 and 1986. Average age of the conifer plantations was ~34 years in 2019. Seedlings of three typical broadleaf tree species, *L. formosana*, *M. maudiae* and *S. superba*, were mixed and planted with 2.5 × 2.5 m spacing in each pure coniferous stand in 2012 and were 7 years old in 2019. The total area is 40 ha. The three broadleaved species are of the same age and mycorrhizal type.

In recent years, *P. massoniana* stands encountered more serious ecological issues relative to the other two stands. Thus, we only chose the *P. massoniana* stands, where the three broad-leaved trees were mixed and planted as a case study. Six hilly slopes of *P. massoniana* stands mixed with the three broad-leaved trees were randomly selected as blocks and were separated by over 30 m. In each block, one healthy plant approximating the mean basal diameter at the height of 0.2 m above the ground for each species was selected as the target tree (Table 1). Samples were collected in April, August and December in 2019. Therefore, six plants were sampled for each species at each sampling time. The net primary productivity was the highest in spring (March–May) and summer (June–August) and became the lowest in winter (December–February, Xu et al. 2017). Luo et al. (2017) found that senescent leaves of *L. formosana* still maintain a positive CO_2_ assimilation even in the later stage (early December) in subtropical regions. Moreover, some studies suggest that the root of deciduous trees still take up nitrogen in winter, even in the leafless period, to ensure the plants’ survival in winter and to provide sufficient nutrients for germination in spring (Silla and Escudero 2003, Andresen and Michelsen 2005, Ueda et al. 2010). Some senescent leaves of *L. formosana* still remained on trees when we sampled in December, implying the continuation of photosynthesis and root activity. Thus, we defined April and August as the rapidly growing season and December as the slowly growing season in our study.

**Table 1:** Family, life form, species code, basal diameter and height (±SE) of the target trees.

Species	Family	Life form	Code	Basal diameter (mm)	Height (m)
*Schima superba*	Theaceae	Evergreen	SS	32.9 ± 2.5	2.7 ± 0.3
*Michelia maudiae*	Magnoliaceae	Evergreen	MM	28.9 ± 2.7	2.7 ± 0.2
*Liquidambar formosana*	Hamamelidaceae	Deciduous	LF	30.5 ± 5.7	2.4 ± 0.4

### Root exudate collection and calculation

A modified culture-based collection trap was used to sample root exudates in the field (Phillips et al. 2008, Yin et al. 2013, He et al. 2021, Sun et al. 2021). First, we carefully excavated the fine roots (≤2 mm in diameter) from the upper 20 cm of undisturbed soil around the target trees and traced the roots back to the parent trees to identify species (Guo et al. 2008). An intact and undamaged fine root system with a length of 20–30 cm (generally having five root orders) was selected and gently washed with a C-free nutrient solution (0.1 mmol L^−1^ KH_2_PO_4_, 0.2 mmol L^−1^ K_2_SO_4_, 0.2 mmol L^−1^ MgSO_4_ and 0.3 mmol L^−1^ CaCl_2_) to minimize the osmotic stress. The intact root system was inserted into a 50-mL glass cuvette, which was then filled with 1 mm of sterile acid-washed glass beads as the growth medium and 10 mL of C-free nutrient solution. The cuvette containing the intact root system was then sealed with Parafilm. Additionally, a moist Kimwipe (Kimberly-Clark Corp., Roswell, New Mexico, USA) was placed around the upper root segment to prevent the exposed portion of the root from drying out. The cuvette system was then covered by plastic bags, returned to the soil and covered with litter to allow the root system to equilibrate with the cuvette environment. One cuvette with no root system filled with sterile glass beads and 10 mL of nutrient solution was prepared as a control in each block. After a 2-day equilibration period, the solution in the buried cuvette was discarded, and the root was flushed five consecutive times with 10 mL of fresh nutrient solution to remove residual C (Phillips et al. 2008, Yin et al. 2013). As soil and litter attached to fine roots (especially, the first-order roots) is difficult to clean completely without damaging the root system, this process can effectively reduce the residual soluble C attached to the roots to ensure that an accurate exudation rate is determined. Ten milliliters of fresh nutrient solution were then added again, and the cuvette was re-buried. After 24 h, the exudates were collected from the cuvette with an automatic electric vacuum pump and were flushed three times with 10 mL of fresh nutrient solution to collect all root exudates. The cuvettes were refilled with 10 mL of fresh nutrient solution and were incubated again for 24 h. The exudates were continuously collected at 24-h interval for 3 days (Phillips et al. 2008, 2011, Yin et al. 2013). The exudation rate for each species did not significantly reduce with sampling time, but it had a large coefficient of variation (CV) (Table S2 available as Supplementary data at *Tree Physiology* Online) when the exudates collected in every time were separately measured, implying that roots still are vigorous at the third sampling time. Thus, the exudates from the three collections were combined and mixed in our formal experiment. In other studies, exudations were also collected at 24-h interval for 3 or even 5 consecutive days (Phillips et al. 2008, 2011, Drake et al. 2011, Yin et al. 2013). The solutions mixed were filtered using 0.45-μm Minisart syringe filters and were then stored at −20 °C. The filtered solutions were analyzed for organic C on a Liqui TOC II analyzer (Elementar, Frankfurt, Germany) within 1 week.

Traditionally, the root exudation rate is calculated in units of whole fine root system (≤2 mm), including the fourth- and higher-order roots (Phillips et al. 2008, Tückmantel et al. 2017, Meier et al. 2020, He et al. 2021). However, absorptive functions of roots are primarily attributed to the most distal roots (Finzi et al. 2015, Freschet et al. 2017), which are more closely related to the root physiological activity and are responsible for acquiring resources. An expression of exudation rate based on the root orders’ performing absorption is of great importance for accurate C and nutrient cycling assessment. In our study, although roots of the first five orders (≤2 mm) were inserted to the exudate collection cuvette, the C accumulation was significantly correlated only with the dry weight of the first-, second- and third-order roots (*P* < 0.05, Figure S1 available as Supplementary data at *Tree Physiology* Online), indicating that exudation is mainly attributed to the roots of the first three orders. Thus, root exudation rate (μg C mg^−1^ day^−1^) was calculated based on the total dry weight of the first- to third-order roots as follows:


(1) \begin{equation*} \text{Root}\ \text{exudation}\ \text{rate}=\frac{1}{3}{\Sigma}_{i=1}^3\frac{{\text{CA}}_{\text{i}}-{\text{CA}}_{\text{CKi}}}{{\text{RDW}}_{1-3}}, \end{equation*}

where CA_i_ (μg C mg^−1^) is the amount of C accumulation in each cuvette containing intact root system on the *i*th day of collection, and CA_CKi_ (μg C mg^−1^) is the amount of C accumulation in each cuvette with no root system on the *i*th day of collection. The RDW_1–3_ (mg) is the total dry weight of the first- to third-order roots. Root exudation rate is averaged over 3 days of collection.

### Root sampling, processing and root morphological and architectural trait assessments

After exudate sampling, the intact fine roots in the cuvette system were harvested and were then dissected with thin forceps according to the hierarchical branching order. The first-order roots are the most distal roots, and the higher-order roots are defined by the connection between two lower-order roots. For example, the connection between two first-order roots are defined as the second-order roots (Pregitzer et al. 2002). The root samples were then placed in deionized water and were scanned by root order using an Epson Expression 10000XL scanner at a resolution of 400 d.p.i. (see more details in Yan et al. 2019, Gao et al. 2021). Afterward, root samples were oven-dried to constant weights at 65 °C and were weighed. Excluding root dry weight, other root morphological and architectural traits were analyzed using a WinRHIZO (Regents Instruments Inc., Quebec, Canada). Here, only the first- to third-order roots were used to coincide with exudation. The morphological traits included RD, RL, root surface area (RS), SRL, SRA and RTD, and the architectural trait was BI. The definitions and calculation methods of root traits described are presented in Table S3 available as Supplementary data at *Tree Physiology* Online.

### Statistical analysis

The relationships between C accumulation in the cuvette and root dry weight of each root order were determined using Pearson’s correlation analysis. The effects of season and species on root traits were tested using linear mixed-effects models (LMM), with season and species as fixed effects and block as a random effect. The CV of each root trait was calculated across species and seasons. Principal components analysis (PCA) was used to determine the pattern of root traits. The relationships between root exudation rate and root morphological and architectural traits across species were identified by standard major axis regression (SMA). The PCA and SMA were performed using the ‘vegan’, and ‘smatr’ packages in R (Warton et al. 2012, Oksanen et al. 2020). Other statistical analyses were conducted using IBM SPSS Statistics version 26.0. The figures were edited, typeset and enhanced using the ‘ggplot2’ package in R (Wickham 2016).

## Results

### Seasonal variations in root traits

**Figure 1:**
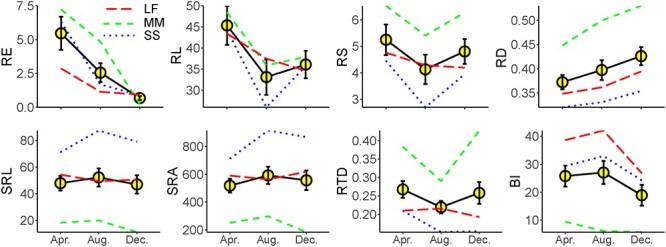
Seasonal variation in the root functional traits of three tree species. RE, RL, RS, RD, SRL, SRA, RTD and BI are root exudation rate (μg C mg^−1^ day^−1^), root length (cm), root surface area (cm^2^), root diameter (mm), specific root length (m g^−1^), specific root area (cm^2^ g^−1^), root tissue density (g cm^−3^) and branching intensity (cm^−1^), respectively. The circle represents the average value of the three species. The data details of each species see Table S4 available as Supplementary data at *Tree Physiology* Online.

**Figure 2:**
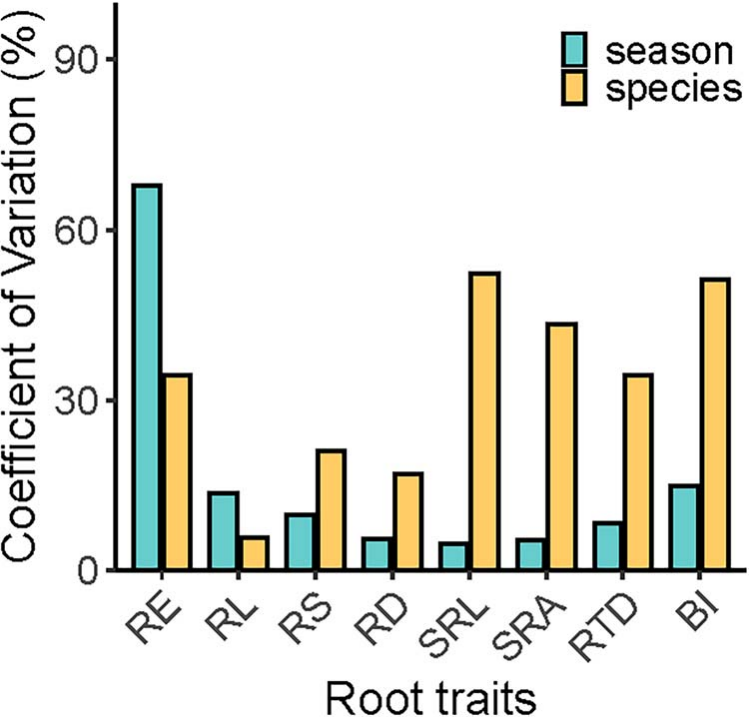
Coefficients of variation of root functional traits across seasons and species. See Figure 1 for the abbreviations.

The RE varied significantly across seasons (*P* < 0.05, Figure 1, Table 2 and Table S4 available as Supplementary data at *Tree Physiology* Online) with a CV of 67.8%, which was larger than the variation across species (CV = 34.5%, Figure 2). The RE was the highest in April and the lowest in December, and it did not significantly vary among species. The RD and RTD varied greatly among seasons (Figure 1 and Table 2), with CVs of 5.5 and 8.4%, respectively (Figure 2). Other traits, excluding RE and RL, were significantly different between species (Table 2). The CV of RE was the highest regardless of across species or seasons (Figure 2).

**Table 2:** *P*-values of the LMMs for the effects of season, species, and their interactions on root functional traits. See Figure 1 for the abbreviations.

Root traits	Source of variation		
	Season	Species	Season × species
RE	**0.001**	0.126	0.332
RL	0.131	0.681	0.919
RD	**0.001**	**0.000**	0.512
RS	0.324	**0.008**	0.945
RTD	**0.013**	**0.000**	**0.002**
SRL	0.196	**0.000**	0.054
SRA	0.082	**0.000**	0.013
BI	0.090	**0.000**	0.617

Bold values denote a significant effect (*P* < 0.05).

### Linkages among root traits

**Figure 3:**
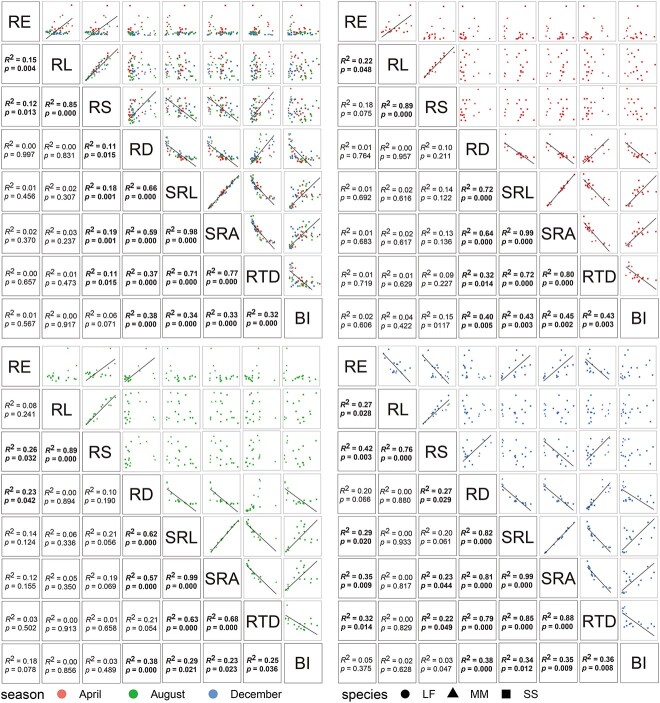
The SMA among eight root functional traits across all species and seasons. See Figure 1 for the abbreviations.

There existed obviously positive correlations among SRL, SRA and BI and between RD and RTD (Figure 3). Meanwhile, SRL, SRA and BI had significant negative associations with RD and RTD. The linkages of RD, SRL, SRA, RTD and BI did not change with growing season, indicating the conservative correlations among them.

The RE was significantly positively related to RL and RS when all data were pooled together (Figure 3), indicating a coordinated correlation between exudates and RL and RS. However, their relationships varied with growing season, in which RE was positively related to RL in April but was negatively related with it in December (Figure 3). Moreover, a positive correlation in August and a negative correlation in December were found between RE and RS. The RE also had a positive correlation with SRL and SRA and had a negative correlation with RTD in December. The result suggested a plastic correlation between RE and other root traits with the growing season.

**Figure 4:**
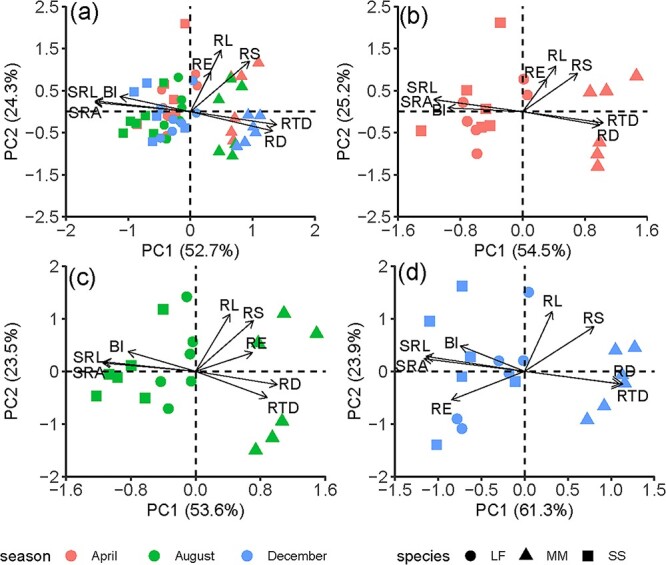
The PCA of eight of the functional traits of roots of the first three orders in (a) all sampling seasons, (b) April, (c) August and (d) December. See Figure 1 for the abbreviations.

The first two axes of Principal components analysis (PCA) accounted for 77–85% of total variation for all species (Figure 4a). Overall the first axis was best described by RD, RTD, SRL, SRA and BI, while the second axis was represented by RE, RL and RS. However, the location of RE shifted with growing seasons. In April and August, RE was loaded similar to RL and RS (Figure 4b and c); however, in December, RE followed the opposite direction from RL and RS (Figure 4d).

## Discussion

### Great seasonal plasticity of root exudation

Root exudates come from the newly assimilated carbohydrates and the stored non-structural carbohydrates of plants, which are passively or actively transported from root cells to the environment through exudation (Gougherty et al. 2018). Higher non-structural carbohydrates in plants should mirror larger root exudation in principle (Prescott et al. 2020), as the photosynthetic capacity and root exudation are closely related (Gougherty et al. 2018). The photosynthetic capacity and non-structural carbohydrates reveal pronounced seasonality (Furze et al. 2019), which might imprint on the seasonality of exudation (Figure 1). Particularly, the CV of exudation was higher across growing seasons than across species (Figure 2), which further reflected that exudation had a higher environmental plasticity. Compared with the root morphological and architectural traits mainly controlled by phylogeny (McCormack et al. 2015, Ostonen et al. 2017, Valverde-Barrantes et al. 2017), root exudation is also subjected to multiple environmental constraints, such as solar radiation (Nakayama and Tateno 2018), temperature (Leuschner et al. 2022), drought (Preece et al. 2018, 2021, Brunn et al. 2022) and soil nutrient availability (Meier et al. 2020, Jiang et al. 2022). Such environmental factors significantly influence the photosynthates and non-structural carbohydrates (Ma et al. 2022), which show remarkable seasonality in subtropical forests (Xu et al. 2017, Yan et al. 2022). Additionally, exudation release could be adjusted quickly, which was also the reason for the strong plasticity. The adjustment of root morphological and architectural traits cost time to grow, while the adjustment of exudation was more rapid and flexible. Studies suggested that root exudation was even affected by day-to-day meteorological changes (Phillips et al. 2008, Sun et al. 2017). Although the exudation was also controlled by the plant itself, the three selected species had consistent mycorrhizal type, tree age and forest stand conditions, which may weaken the difference in exudation and strategies among the three species. Relatively, the variation across species was lower than that across seasons.

Plants tend to devote a large amount of energy to nutrient acquisition organs in the early growing season (Eissenstat and Yanai 1997) because the suitable temperature and precipitation (Table S1 available as Supplementary data at *Tree Physiology* Online) facilitate rapid plant growth (Meng et al. 2021), and thus promote the high nutrient requirements of plants. More photosynthetic production and greater nutrient requirements may generate a higher exudation rate (Phillips et al. 2008, Wang et al. 2021). Obviously, plants also exuded the highest C in April (Figure 1 and Table 2), which was consistent with the finding by Phillips et al. (2008) that roots exude more at the onset of growth. Meanwhile, abundant precipitation in April may cause hypoxia of the root system (Badri and Vivanco 2009) and can lead to the accumulation of ethanol and lactic acid (Rivoal and Hanson 1994). Plants would release these toxic substances to avoid injuries in the form of exudates (Xia and Roberts 1994), which also result in the increases in amounts of root exudation.

Traditionally, plants also need a large amount of nutrients to meet their vigorous growth in August (Xu et al. 2017). It seems that plants would release more exudates to fuel microbes for nutrient exchange. However, it is surprising that root exudation rate reduced in August compared with in April (Figure 1). First, the increase in SRL and the decrease in RTD (Figure 1) reflect a greater root absorption capacity and root activity of plants (Kramer-Walter et al. 2016, Weemstra et al. 2016). Second, mycorrhizal associations are one of the key ways for plants to acquire resources (Eissenstat et al. 2015, Chen et al. 2016, Liu et al. 2020). Arbuscular mycorrhizal production is higher in August in subtropical China due to warming (Jiang et al. 2022), which induces a higher nitrogen uptake rate (Liu et al. 2020). Thus, plants tend to acquire more nutrients through mycorrhizal associations than through exudation in August, which may reduce root exudation. This is supported by the finding of Meier et al. (2020) that root exudation is closely negatively linked to fungal abundance and activity. Third, the dry seasons last up to 4 months (Table S1 available as Supplementary data at *Tree Physiology* Online), which possibly reduces root exudation. Contrary to the finding that drought generally stimulates more root exudation (Eziz et al. 2017, Preece et al. 2018, Qiao et al. 2022), long-term drought would decrease photosynthesis, thereby reducing plant C rhizodeposition (Hasibeder et al. 2015, Holz et al. 2018, Chomel et al. 2019, Williams and de Vries 2020). Additionally, root exudation was lower based on the individual root system but was higher based on the whole-tree level, possibly due to higher root biomass. Nutrient requirements of plants could be met partly through whole-tree-level exudation (Brunn et al. 2022).

Plants would decrease their demands for nutrients due to the slow growth in December (Xu et al. 2017), which reduces their investment in exudation (Figure 1). Additionally, plants may allocate less C to belowground in winter due to slow photosynthesis induced by the colder temperatures, less rainfall (Figure S1 available as Supplementary data at *Tree Physiology* Online) and the lower photosynthetically active radiation (Meng et al. 2021).

### Seasonal changes in the relationships among root exudates with other root traits

Root functional traits may be coordinated or traded off at different scales (Roumet et al. 2016, Ma et al. 2018, Wen et al. 2019). The trade-off of SRL and SRA with RD did not change with sampling season (Figure 3) because most root traits are mainly controlled by phylogeny (McCormack et al. 2015, Ostonen et al. 2017, Valverde-Barrantes et al. 2017). However, RE was extremely sensitive to sampling season (Figure 1), indicating that the relationships between RE and other root traits seasonally varied (Figures 3 and 4). Thus, considering the similarity of the three species we selected, and the great seasonal plasticity of root exudation, the analysis of relationships is based on plant individuals rather than species.

Plants grow rapidly in April and August (Xu et al. 2017, Meng et al. 2021) when they require large amounts of nutrients. Plant individuals with larger total RL and total surface area may grow faster and require more nutrients, or may have more surplus C to release because of sufficient photosynthetic products, which is in accordance with our hypothesis.

A higher RTD generally suggests higher tissue investment (Weigelt et al. 2021), indicating that roots would spend more C building the same size roots compared with a lower RTD. In December, the investment in exudation reduced but increased for maintaining; thus, a trade-off was observed between RE and RTD (Figure 3), which is consistent with our hypothesis. Interestingly and importantly, a significant positive relationship was observed between RE and SRL and SRA in December; however, no significant relationships were observed in April and August in our study (Figures 3 and 4). The SRL and SRA reflect the root uptake area at a given biomass cost, which indicates the nutrient acquisition capacity (Weemstra et al. 2016). However, root exudates are not necessarily used for nutrient acquisition purposes but may also be a way to remove surplus C (Prescott et al. 2020). During the rapid growing season, ethanol and lactic acid increased by excessive soil moisture (Rivoal and Hanson 1994, Xia and Roberts 1994, Badri and Vivanco 2009) and surplus C is also released in the form of exudation. Hence, the part of exudation that is not used for nutrient acquisition resulted in a non-significant relationship between RE and other nutrients acquisition root traits at the rapidly growing seasons. Akatsuki and Makita (2020) found that there was no significant relationship between RE and SRA. In December, although belowground C allocation may be reduced by the decline in photosynthates (Xu et al. 2017) due to drought and cold (Table S1 available as Supplementary data at *Tree Physiology* Online), the C may still allow plants to grow slowly (Meng et al. 2021). The senescent leaves of *L. formosana* do not completely shed and still have the photosynthetic capacity in December in the subtropical region (Zhang et al. 2013, Luo et al. 2017), although it is indeed a deciduous species. Additionally, even if no photosynthesis occurred in winter due to leaflessness, the starch reserves in plants would break down (Wargo 1979) and the resulting soluble sugars would be allocated for root elongation and maintenance of the belowground system (Kozlowski 1971). Previous studies also mentioned that deciduous trees could absorb nutrients even during the leafless period in winter (Silla and Escudero 2003, Andresen and Michelsen 2005, Ueda et al. 2010). Therefore, *L. formosanais* would still release root exudates like evergreen species in December to sustain the plant activity and growth, although the amount would be small. Without surplus C releasing, the exudations were released purposefully for obtaining nutrients, resulting in the markedly positive relationships between RE, and SRL and SRA in December.

## Conclusion

The root exudation rate was a more plastic trait than morphological and architectural traits. We observed strong seasonality in the root exudation rate, which induced their various relationships with other root traits at different growing seasons. The rapid adjustment and great plasticity of root exudation may render it the most important root trait for plant adaptation. However, the large variability of root exudation also increased the complexity of the linkages between exudation and other root traits. Further investigations of more species during different growing seasons are needed to better elucidate the role of root exudates in the root economic spectrum.

## Supplementary Material

Supplementary_material_clean_editionv5_tpad118

## Data Availability

The data are available at https://www.scidb.cn.
