# Supplementary material for: Relationships between root exudation and root morphological and architectural traits vary with growing season
Source: Tree Physiol. 2023 Sep 21;44(1):tpad118. doi: 10.1093/treephys/tpad118 (PMC10849755; doi:10.1093/treephys/tpad118)
Supplement: Supplementary_material_clean_editionv5_tpad118 [file supplementary_material_clean_editionv5_tpad118.docx]

Table S1 Monthly mean temperature and precipitation at the QYZ site in 2019

|  | Spring | | | Summer | | | Autumn | | | Winter | | |
| --- | --- | --- | --- | --- | --- | --- | --- | --- | --- | --- | --- | --- |
| Month | Mar. | Apr. | May | Jun. | Jul. | Aug. | Sep. | Oct. | Nov. | Dec. | Jan. | Feb. |
| TEMP  (°C) | 13.8 | 20.3 | 22.1 | 26.3 | 27.6 | 29.2 | 25.6 | 20.7 | 14.3 | 9.1 | 7.2 | 8.0 |
| P (mm) | 155.2 | 207.2 | 164.6 | 313.2 | 232.0 | 45.4 | 14.2 | 34.2 | 2.4 | 49.6 | 63.2 | 150.2 |

Table S2 An excerpt of unpublished data on the rate of exudation (μg C mg^−1^ d^−1^) among 3 days in the previous experiment (carbon of blank control have been removed)

|  | Day-1 | Day-2 | Day-3 |
| --- | --- | --- | --- |
| *Cunninghamia lanceolata* | 0.11 | 0.27 | 0.35 |
| *Liriodendron chinense* | 3.34 | 3.11 | 1.64 |
| *Toona sinensis* | 2.30 | 1.40 | 0.68 |
| *Choerospondias axillaris* | 3.72 | 3.11 | 2.99 |
| *Castanea mollissima* | 3.62 | 1.60 | 1.57 |
| *Pinus elliottii* | 0.91 | 0.44 | 1.08 |
| *Cinnamomum porrectum* | 2.87 | 7.33 | 2.23 |
| *Diospyros lotus* | 3.25 | 4.20 | 1.33 |
| *Castanopsis fargesii* | 9.41 | 7.79 | 3.85 |
| *Castanopsis eyrei* | 12.21 | 8.29 | 8.90 |

Table S3 Definitions and descriptions of fine-root traits referred by Yan, et al. (2019) and Gao et al. (2021).

| Root trait | Abbreviation | Unit | Description |
| --- | --- | --- | --- |
| **Physiological trait** |  |  |  |
| Root exudation rate | RE | μg C mg^−1^ d^−1^ | The rate of C exudation per day measured by dry weight of absorptive roots |
| **Morphological trait** |  |  |  |
| Root diameter | RD | mm | Average root diameter of roots |
| Root length | RL | cm | total root length of each order absorptive roots |
| Root surface area | RS | cm^2^ | total root surface area of each order absorptive roots |
| Specific root length | SRL | m g^-1^ | The ratio of root cluster length to root dry mass |
| Specific root area | SRA | cm^2^ g^-1^ | The ratio of root cluster area to root dry mass |
| Root tissue density | RTD | g cm^-3^ | The ratio of root dry mass to root volume |
| **Architectural trait** |  |  |  |
| Branching intensity | BI | cm^-1^ | Ratio of number of first- and second-order roots to length of third-order roots |

Table S4 The average and coefficient of variation (CV) of the root functional traits of the tree species in April, August, and December. RE, RL, RS, RD, SRL, SRA, RTD, and BI are root exudation rate (μg C mg^−1^ d^−1^), root length (cm), root surface area (cm^2^), root diameter (mm), specific root length (m g^−1^), specific root area (cm^2^ g^−1^), root tissue density (g cm^-3^), and branching intensity (cm^−1^), respectively. Total is all three species, SS is *S. superba*, MM is *M. maudiae*, and LF is *L. formosana*.

| season | species | RE | | RL | | RS | | RD | | SRL | | SRA | | RTD | | BI | |
| --- | --- | --- | --- | --- | --- | --- | --- | --- | --- | --- | --- | --- | --- | --- | --- | --- | --- |
|  |  | AVE | CV(%) | AVE | CV(%) | AVE | CV(%) | AVE | CV(%) | AVE | CV(%) | AVE | CV(%) | AVE | CV(%) | AVE | CV(%) |
| Apr. | SS | 6.3 | 113.0 | 44.3 | 41.3 | 4.5 | 42.7 | 0.3 | 2.0 | 71.0 | 15.6 | 712.5 | 15.8 | 0.2 | 19.2 | 29.3 | 18.0 |
| Apr. | MM | 7.2 | 58.1 | 48.4 | 42.6 | 6.5 | 40.5 | 0.5 | 11.4 | 18.3 | 17.5 | 251.0 | 16.0 | 0.4 | 20.0 | 9.4 | 39.8 |
| Apr. | LF | 2.9 | 49.9 | 43.4 | 45.3 | 4.8 | 46.8 | 0.4 | 2.2 | 54.3 | 7.6 | 590.2 | 7.6 | 0.2 | 12.4 | 38.6 | 42.3 |
| Aug. | SS | 1.7 | 27.9 | 26 | 63.0 | 2.7 | 63.0 | 0.3 | 2.0 | 87.3 | 14.1 | 914.0 | 13.5 | 0.2 | 10.5 | 32.9 | 16.2 |
| Aug. | MM | 4.9 | 86.8 | 35.9 | 50.7 | 5.4 | 46.0 | 0.5 | 16.2 | 19.9 | 28.8 | 298.0 | 21.4 | 0.3 | 25.1 | 6.1 | 36.7 |
| Aug. | LF | 1.1 | 65.9 | 37.5 | 43.9 | 4.3 | 46.0 | 0.4 | 2.5 | 49.3 | 8.3 | 563.2 | 8.5 | 0.2 | 13.3 | 42.0 | 33.9 |
| Dec. | SS | 0.9 | 60.7 | 35.8 | 39.8 | 3.9 | 37.7 | 0.4 | 3.0 | 79.0 | 18.7 | 867.6 | 17.6 | 0.2 | 25.3 | 24.2 | 76.1 |
| Dec. | MM | 0.2 | 54.7 | 37.9 | 23.2 | 6.3 | 25.2 | 0.5 | 7.0 | 11.2 | 11.8 | 184.9 | 9.5 | 0.4 | 11.3 | 5.7 | 36.4 |
| Dec. | LF | 0.9 | 75.6 | 34.6 | 49.0 | 4.2 | 47.5 | 0.4 | 4.9 | 50.5 | 15.0 | 617.8 | 14.4 | 0.2 | 7.4 | 26.8 | 46.8 |


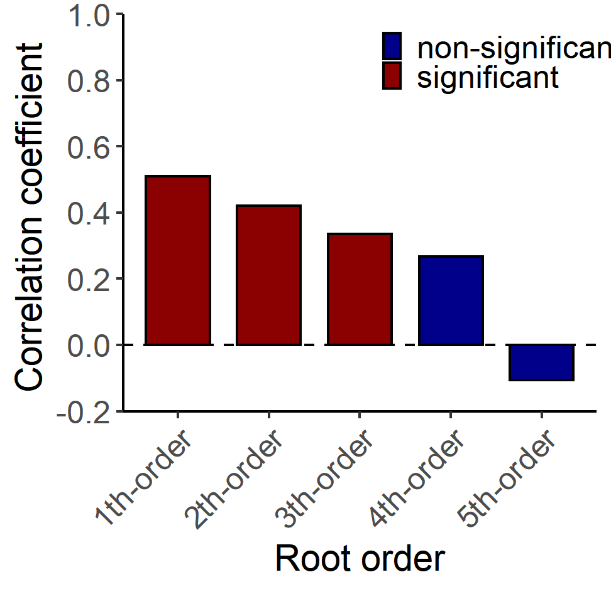


Figure S1 Correlation coefficient between C accumulation and the dry weight of roots of each order in the exudate collection trap.
